# Differentiation of patient-specific void urine-derived human induced pluripotent stem cells to fibroblasts and skeletal muscle myocytes

**DOI:** 10.1038/s41598-023-31780-9

**Published:** 2023-03-23

**Authors:** M. Kibschull, T. T. N. Nguyen, T. Chow, M. Alarab, S. J. Lye, I. Rogers, O. Shynlova

**Affiliations:** 1 Lunenfeld-Tanenbaum Research Institute, Sinai Health System, 25 Orde St, Suite 6-1017, Toronto, ON M5T 3H7 Canada; 2 Department of Physiology, University of Toronto, Toronto, Canada; 3 Department of Obstetrics and Gynecology, University of Toronto, Toronto, Canada; 4 Division of Urogynecology and Reconstructive Pelvic Surgery, Mount Sinai Hospital, Toronto, Canada

**Keywords:** Cell biology, Molecular biology, Urology

## Abstract

Cell-based therapy is a major focus for treatment of stress urinary incontinence (SUI). However, derivation of primary cells requires tissue biopsies, which often have adverse effects on patients. A recent study used human induced pluripotent stem cells (iPSC)-derived smooth muscle myocytes for urethral sphincter regeneration in rats. Here, we establish a workflow using iPSC-derived fibroblasts and skeletal myocytes for urethral tissue regeneration: (1) Cells from voided urine of women were reprogrammed into iPSC. (2) The iPSC line U1 and hESC line H9 (control) were differentiated into fibroblasts expressing FSP1, TE7, vinculin, vimentin, αSMA, fibronectin and paxillin. (3) Myogenic differentiation of U1 and H9 was induced by small molecule CHIR99021 and confirmed by protein expression of myogenic factors PAX7, MYOD, MYOG, and MF20. Striated muscle cells enriched by FACS expressed NCAM1, TITIN, DESMIN, TNNT3. (4) Human iPSC-derived fibroblasts and myocytes were engrafted into the periurethral region of RNU rats. Injected cells were labelled with ferric nanoparticles and traced by Prussian Blue stain, human-specific nuclear protein KU80, and human anti-mitochondria antibody. This workflow allows the scalable derivation, culture, and in vivo tracing of patient-specific fibroblasts and myocytes, which can be assessed in rat SUI models to regenerate urethral damages and restore continence.

## Introduction

Pelvic floor disorders (PFDs) are a hidden epidemic among women of all ages. Two major PFDs, stress urinary incontinence (SUI) and pelvic organ prolapse (POP) impact 30–50% of older women, affecting their quality of life^1^. By age 80, up to 20% of all women will have had a reconstructive pelvic surgery for one of these conditions. At least 30% of these patients will require reoperation^2^. SUI, the involuntary loss of urine during physical effort, could be a consequence of childbirth injury to the urethral sphincter, which intensifies with age and the onset of menopause^3^. SUI is primarily due to either intrinsic sphincter deficiency (ISD)^4^, or urethral hypermobility caused by the lack of structural support of the surrounding pelvic floor tissues^5^. ISD is usually caused by injury to either smooth or striated muscle layers of the urethral sphincter, or to its innervation, which all can contribute to the development to SUI^6–8^. Current clinical options for SUI use mechanical support of the affected organ by minimally invasive surgery: synthetic mid-urethral sling to lift the urethra, or office-based procedures such as periurethral injection of bulking agents^9^. These are gold standard in many surgeons’ opinion, as multiple studies of short and long-term outcome data showed satisfactory results^4^. However, these treatment options are suboptimal, as none of these methods can fully restore normal sphincter function^9^, but rather substitute for the damaged and atrophied tissue with an inert substance, and are associated with known complications^10,11^. Moreover, several international regulatory bodies have challenged and placed moratoriums on use of any transvaginal mesh product including mid-urethral sling until further clarity regarding the long-term risk/benefit profile is established^10^. There is an urgent need to develop alternatives for urethral sphincter regeneration, to help women suffering from this disabling condition.

Cell-based therapies and tissue engineering for the regeneration of injured urethral tissues have potential as a durable treatment option for SUI^12^. They promise full reversal of the primary pathophysiology of intrinsic urethral sphincter deficiency. Selecting the appropriate cell type for urological tissue regeneration is an important factor for in vivo graft survival and function. An ideal source of cells should be easily accessible, abundant, and available from all patients^13^. Stem cells (SCs) are an invaluable tool for promoting regrowth of diseased tissue as they self-renew and have long-term viability. A large number of adult SCs have been described for SUI treatment: mesenchymal SCs (MSCs), adipose-derived MSCs, bone marrow-derived, endometrial MSCs, and muscle-derived SCs^14^. However, these cell-based therapies require invasive biopsies (i.e., bone marrow punctation or liposuction under general/local anesthesia). Growing evidence indicates that autologous muscle-derived cells represent a potential therapeutic option for augmentation of urethral sphincter function^15–17^. A clinical study showed that treatment of female SUI by peri- or intraurethral injections of in vitro expanded autologous muscle-derived primary cells (myocytes and fibroblasts) resulted in enhanced continence or improved symptoms 1–2 years after injections^15,17^. Multiple international clinical trials in both, male and female patients were initiated to investigate use of autologous muscle-derived cells, although the invasive muscle biopsy collection method, the wide variations in cell isolation and cultures techniques, dosing, and measured efficacy parameters prevented adequate analysis of these data^12^.

To circumvent the problems associated with tissue biopsy and establishment of primary cultures, we intend to produce autologous cells generated non-invasively from patient-specific induced pluripotent stem cells (iPSCs). Recently, it has been demonstrated that human urine contains a mixture of viable cells (i.e., multipotent MSCs and renal epithelial cells), obtainable in sufficient numbers from a single void, which can be reprogrammable into iPSCs^18^. Furthermore, stable iPSC lines, regardless of the source of origin have similar pluripotent properties and differentiation capacities. Thus, we hypothesize that patient-specific iPSCs-derived from somatic cells in voided urine may represent a novel source for future autologous cell treatment of SUI. Therefore, we aim (1) to generate and characterize iPSC lines derived from viable cells in voided urine of female patients via integration-free episomal reprogramming, (2) to differentiate these iPSC into fibroblasts and skeletal muscle cells, (3) to engraft and trace these cells into the periurethral region of immune-deficient rats.

## Materials and methods

### Ethics

All experiments were performed in accordance with relevant guidelines and regulations: Patients were recruited at the Division of Urogynecology, Mount Sinai Hospital, Toronto, Canada. The study was approved by the Mount Sinai Hospital Research Ethics Board (MSH-REB, #15-0138-E) and informed written consent was obtained. Derivation and use of iPSC lines and the use of the hESC line H9 was approved by the Canadian Stem Cell Oversight Committee and the MSH-REB (#21-0076-E). The use of the H9 line was licensed by WiCell (Madison, USA). Mouse and rat cell engrafting experiments were performed under approved animal use protocols (AUP) by the University Health Network Animal Care Committee (AUP #6244) and the Mount Sinai Hospital Animal Care Committee (AUP #0241H).

### Patient recruitment and urine cell collection

Void urine samples from SUI patients were collected during regular office visits. Samples were immediately spun for 5 min at 500 × g. Pelleted cells were resuspended in Renal Epithelial Cell Medium (ATCC, USA) without antibiotics, seeded into uncoated culture plates, and grown until 90% confluence. Cells were cryopreserved or immediately used for reprogramming.

### iPSC reprogramming and pluripotent stem cell culture

Proliferating urine-collected cells (1*10^5^–1*10^6^) were transfected by electroporation using the Epi5™ system of episomal vectors (Invitrogen, Canada), carrying the reprogramming factors, Kld4, Lin28, L-myc, Oct4, Sox2, and p53 short-hairpin RNA. Electroporation settings and culture conditions for iPSC colony formation were described recently^19^. After 17–21 days, emerging iPSC colonies were picked and individually sub-cultured in vitronectin-coated 24-well plates (Stem Cell Technologies, Canada) in mTeSR1 medium (Stem Cell Technologies) for 5–10 passages. Episomal vector clearance from the iPSC lines was confirmed by PCR as described previously^19^. Episome-free iPSC lines were expanded in mTeSR1 medium in culture flasks coated with Geltrex (Invitrogen), cryopreserved, and characterized for the presence of pluripotency markers by RT-qPCR and immunocytochemistry.

### Embryoid body formation assay and fibroblast differentiation

Fibroblast differentiation of iPSCs was achieved by embryoid body (EB) formation assay. EBs of uniform size were generated from 1*10^6^ iPSCs as described previously^20^ using AggreWell400 plates and AggreWell EB Formation Medium (Stem Cell Technologies). After 3 weeks of suspension culture in E6 medium containing: DMEM/F12 (Invitrogen), 25 µg/mL recombinant human insulin, 10.7 µg/mL human holo-transferrin, 0.5 mg/mL sodium bicarbonate, 64 µg/mL L-ascorbic acid, 14 ng/mL sodium selenite (all Sigma, Canada), supplemented with 10 ng/mL bFGF (Peprotech, Canada) and 100 U/mL penicillium, 100 µg/mL streptomycin (Invitrogen), EBs were dissociated with TrypLE (Invitrogen) and plated in recombinant human Collagen IV-coated (Peprotech) culture dishes. Outgrowing cells were dissociated with TrypLE and replated in chemically defined fibroblast culture medium supplemented with bFGF, until confluent, passaged for five passages, characterized and cryopreserved.

### Myofibroblast differentiation assay

80,000 dissociated fibroblasts were seeded in E6 medium with 10 ng/mL bFGF in uncoated or collagen IV coated 24-well plates. 24 h after seeding, medium was replaced by E6 plus 10 ng/mL bFGF only (control condition), or E6 medium + 10 ng/mL bFGF and 2 ng/mL TGF-β1 (R&D Systems, Canada) (stimulation condition). The plates were cultured for 7 days with daily medium changes. Cells were then fixed with 50/50% methanol/acetone and subjected to immunocytochemistry with an αSMA (DAKO, USA) antibody, and counterstained with DAPI. Per condition, a random picture was taken in the center of four individual 24-wells. The number of αSMA positive myofibroblasts and the total number of nuclei (DAPI) was counted for each well. 

### Statistical analysis

The relative percentage of myofibroblasts in the treatment groups as compared to controls was assessed by Student’s t-test. A *p* value of < 0.05 was considered as statistically significant.

### Myocyte differentiation from pluripotent stem cells

U1 or H9 cells were seeded into Geltrex-coated 12-well plates at a density of 1*10^5^ cells/well in mTeSR1 medium with 10 µM ROCK-inhibitor. The following day (day 0), medium was replaced with E6 medium supplemented with 4 µM CHIR99021 (Sigma) for mesodermal induction. Cells were cultured in E6 + CHIR99021 until day 4, with a medium change at day 2. At day 4, medium was replaced with E6 + 10 µg/mL bFGF and cultured until day 40 with medium changes every other day. At day 40, cells were dissociated using TrypLE Express and passaged at 1:4 dilutions and aliquots cryopreserved in mFreSR (Stem Cell Technologies). After day 40 (passage 1) cells were cultured in E6 alone for myogenic maturation. Cultures could be passaged at 1:4 ratios every 3–4 weeks and maintained for more than 135 days (passage 5). For RNA analysis, one 12-well was lysed in RNeasy Plus buffer (Qiagen, Canada) and processed as described^20^. For immunocytochemistry, one 12-well was split 1:8 into 4-well plates (Nunc, Canada), cultured for 7 days and fixed for double-immunocytochemistry using myogenic lineage markers and appropriate secondary antibodies.

### Myocyte enrichment

Four 12-wells of myocyte cultures were washed with DPBS and incubated with 1 mL of collagenase type IV solution (Stem Cell Technologies) for 20 min at 37 °C. Cells were dissociated by repetitive gentle pipetting and transferred to 5 mL of E6 medium, 1% human serum (Wisent, Canada). After centrifugation at 200 × g for 5 min, cells were resuspended in 400 µl DPBS with 1% HSA (human serum albumin, Sigma) and filtered through a 35 µm cell strainer into a flow tube (Falcon, Canada). 300 µL of cell suspension were incubated with 30 µl NCAM(CD56)-PE-Cy7 and 25 µL of HNK-1(CD57)-PE antibody. In parallel, 30 µl controls of unstained cells, 3 µL NCAM(CD56)-PE-Cy7 only, or 2.5 µl HNK-1(CD57)-PE antibody alone, were prepared. After 30 min incubation in ice, 4 mL of DPBS was added, cells spun for 5 min, and pellets resuspended in 300 µL DPBS with 1% HSA, 10 µM ROCK inhibitor, 25 mM HEPES, and 0.4 µg/mL DAPI. Dead cells take up DAPI, whereas viable cells with intact membranes remain unstained. Cell sorting was performed on a MoFlo Astrios EQ instrument equipped with 355, 405, 488, 561 and 642 nm lasers (Beckman Coulter, Miami, USA). Sorted cells were plated in E6 medium, 10 µg/mL bFGF, 10 µM ROCK inhibitor on Geltrex coated 12-wells, cultured to confluency and expanded over 5 passages by 1:4 splitting. At P5, myocytes were characterized and cryopreserved.

### Immunocytochemistry

iPSCs were seeded into Geltrex-coated 4-well plates (Nunc), cultured until 70% confluence, and fixed with 10% formalin for 15 min at room temperature (RT). Plates were washed and blocked with 10% FBS in DPBS for 30 min at RT. Cells were incubated over night with primary OCT4, SOX2 or NANOG antibodies diluted in 0.2% FBS, 0.1% Triton-X100 in DPBS at 4 °C. All primary and secondary antibodies and utilized dilutions are listed in Supplemental Table S1. Fibroblast and myocyte cultures were fixed with a 50:50% acetone/methanol solution for 5 min at RT, washed, and blocked with 1% BSA, 0.1% Triton-X100 in DPBS for 30 min at RT. Primary antibodies were applied overnight in 1% BSA, 0.1% Triton-X100 in DPBS at 4 °C. After primary antibody incubation, cells were washed, labeled with appropriate secondary antibodies for 45 min at RT, washed, and counterstained with DAPI nuclear stain. Images were taken on a Leica Spinning Disc Confocal Microscope using Velocity software (Teledyne Photometrics, USA).

### Reverse-transcription quantitative PCR

RNA isolation from cell cultures, reverse transcription and RT-qPCR analysis were performed as described previously^20^. The expression of 31 pluripotency associated genes was quantified using assay primers from the PrimePCR human embryonic stem cell panel (Bio-Rad, Canada) with reference targets HPRT-1, B2M, GAPDH, TFRC and TBP (Supplemental Table S2). The expression of myogenic differentiation markers was quantified using reference targets β-actin, HPRT-1, SDHA and YWHAZ (Supplemental Table S2). Data were analyzed using the CFX manager software (Bio-Rad) using the 2^−ΔΔCq^ method.

### Karyotype analysis

iPSC cultures were sent to The Centre for Applied Genomics at the Hospital for Sick Children (Toronto, Canada) for karyotyping. Five metaphase cells from a 6-well dish were counted and assessed via G-banding analysis.

### Western blotting

Protein lysates (50 µg) were separated by electrophoresis using a 12% polyacrylamide gel and blotted onto PVDF membranes (Bio-Rad). Membranes were then incubated over night with primary antibody at 4 °C: HNK-1, NCAM1, ERK (Supplemental Table S1). After 3 washes, membranes were incubated with a horseradish peroxidase-conjugated secondary antibody, developed by Luminata ECL reagent (Millipore), and visualized on a Chemidoc instrument (Bio-Rad).

### Teratoma formation and immunodetection

Injection of U1 iPSC for teratoma formation in mice was performed as described previously^19^. After 12 weeks of growth, tumors were dissected, formalin fixed, paraffin embedded, and submitted to routine immunohistochemistry. Trilineage-specific primary anti-human antibodies TUBB3, HNF-3β, αSMA were used (Supplemental Table S1). Sections were counterstained with hematoxylin, cover slipped and imaged.

### In vivo tracking of iPSC-derived human fibroblasts and myocytes in nude rats

12-week-old, athymic, immunocompromised female nude rats (RNU, NIH-Foxn1*rnu* athymic) were purchased from Charles River Laboratories (USA). Animals were housed at the UHN animal facility in a pathogen-free environment with a fixed 12 h light/dark cycle. Handling of rats and experiments were in accordance with ARRIVE guidelines (https://arriveguidelines.org). 24 h before injection, U1 fibroblasts were labelled in vitro by adding 30 µg/mL iron oxide nanoparticle colloid Molday ION Rhodamine B (BioPAL, USA) to the culture medium. Fibroblasts or myocyte cultures were dissociated using TrypLE (Invitrogen), twice washed with E6 medium and resuspended in 150 µl E6 medium + 100 ng/mL bFGF. Rats were anesthetized using isoflurane and 100 µl of the cell suspension (2*10^6^ cells) was injected into the periurethral area of rats using a 25G5/8 needle (two injections at 3 and 9 o’clock positions, 1*10^6^ cells each). Three weeks post-injection, rats were euthanized, the urogenital tract tissues dissected, formalin fixed, and paraffin embedded.

### Histology and immunocytochemistry

Cross-sections of rat urogenital tissues were stained for H&E and Masson’s-trichrome to assess tissue morphology. Human fibroblasts or myocytes, labelled with the iron oxide nanoparticle colloid Molday ION Rhodamine B, were identified in the rat tissues using Prussian Blue staining. Briefly, tissue blocks were sectioned at 7 µm, mounted onto Super Frost slides (Thermo Fisher, Canada), de-paraffinized, and rehydrated. Slides were washed for 5 min in distilled water and then incubated for 20 min at RT in Perl’s solution (1% potassium ferrocyanide (Sigma), 1N hydrochloric acid (Fisher Scientific)). After blue color development, slides were rinsed in distilled water, counterstained with 1% Nuclear Fast Red (Electron Microscopy Technology, Canada), dehydrated and cover slipped. In addition, engrafted human cells within rat tissues, were detected using antibodies targeting human-specific nuclear protein KU80, and human mitochondria (hMitochondria, Supplemental Table S1). Visualization was performed using a biotinylated anti-mouse secondary antibody (DAKO, or a biotinylated anti-rabbit secondary antibody (Vector Labs, USA), followed by streptavidin (Invitrogen, 1:2000) binding, and DAB (DAKO) colorimetric development. Sections were counterstained with hematoxylin and cover slipped. Images were captured using a research slide scanner and the OlyVIA software (Olympus, Canada).

## Results

### Derivation and characterization of iPSC from void urine cells

**Figure 1:**
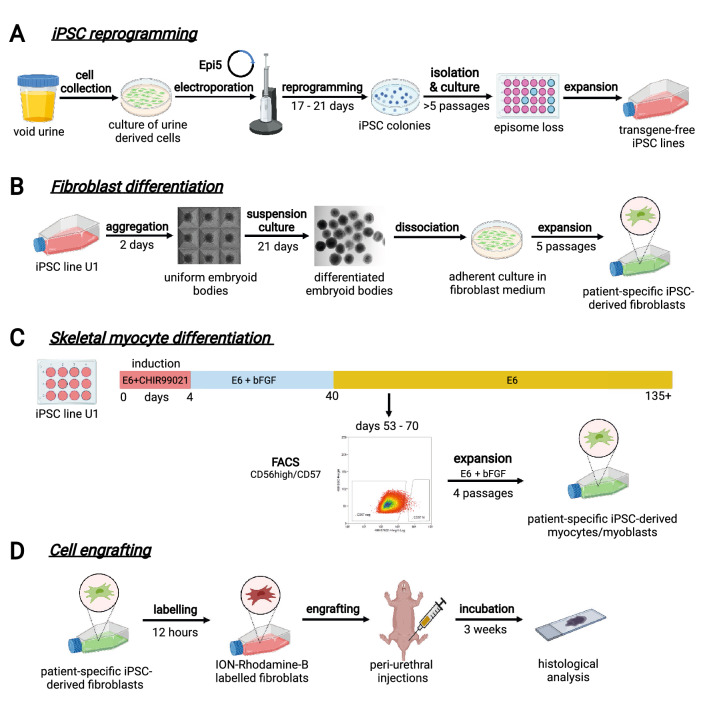
Workflow charts of experimental design. (**A**) *iPSCs reprogramming from urine samples*. Cells collected from void urine samples of SUI patients were cultured and then electroporated with the Epi5™ episomal reprogramming system. After 17–21 days of culture, emerging iPSC colonies were isolated and individually cultured. During > 5 passages of iPSC sub-culture, episomal reprogramming factors were lost and growth of patient-specific, transgene-free iPSC lines is maintained by endogenous pluripotency markers. (**B**) *Fibroblast differentiation*. A single-cell suspension of iPSC was spun into AggreWell multi-well plates to form embryoid bodies (EBs). After 2 days, EBs were transferred to suspension culture dishes for 21 days to induce spontaneous differentiation. EBs were then dissociated, and cells transferred to adherent culture dishes in fibroblast growth medium. After 5 passages, cultures were tested for fibroblast marker expression and purity, expanded, and cryo-preserved. (**C**) *Skeletal myocyte differentiation.* iPSCs were seeded in 12-well plates. Mesodermal differentiation was induced by culturing iPSC in E6 medium supplemented with small molecule CHIR99021 for 4 days. Subsequent culture in E6 medium and bFGF led to myogenic cell differentiation and expansion. From day 40 onwards, cells were maintained in E6 medium alone, leading to myocyte differentiation and myotube formation. Between days 53–70, culture samples were dissociated and CD56^high^/CD57^neg^ myocytes isolated by FACS. Enriched myocyte cultures were expanded for four passages, and cryopreserved. (**D**) *Cell engrafting in rat tissues and histological assessment.* Cultures of patient-specific iPSC-derived fibroblasts were labelled with Molday ION Rhodamine B nanoparticles for in vivo cell tracing. Suspensions of labelled fibroblasts were injected into the peri-urethral region of immune-compromised female nude RNU rats. After 3 weeks, urogenital tissues were dissected, fixed, processed, and histologically analyzed.

**Figure 2:**
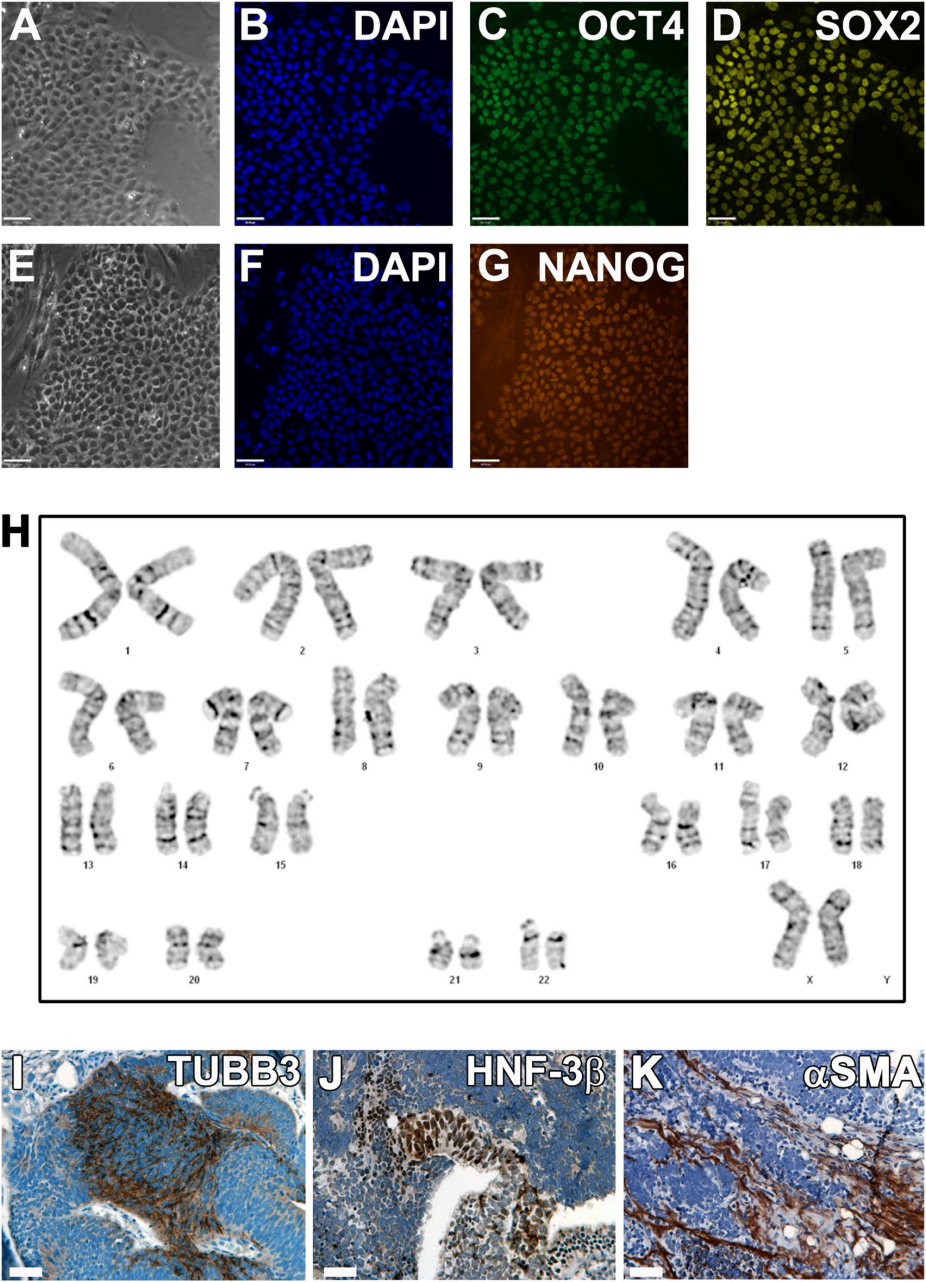
Characterization of urine cell-derived iPSC line U1. Urine cells were reprogrammed to iPSCs using the Epi5 reprogramming system. (**A**–**G**) Immunocytochemistry of episome-free U1 line confirms the expression of pluripotency markers in the newly derived iPSC line U1: (**A**) phase contrast image of a U1 iPSC colony grown on Geltrex in mTeSR, and corresponding co-immunolabeling for (**B**) DAPI, (**C**) OCT4, and (**D**) SOX2. (**E**) Phase contrast, and co-immunolabeling for (**F**) DAPI, and (**G**) NANOG. Bar: 100 µm. (**H**) G-banded analysis of U1 metaphase chromosomes reveals a normal female karyotype. (**I**, **J**, **K**) Teratoma formation assay of undifferentiated U1 injected into kidney capsule of immune deficient athymic nude mice. Immunohistochemistry of dissected tumors shows tri-lineage differentiation into TUBB3 positive neuroectoderm (**I**, brown staining), hepatocyte nuclear factor HNF-3β expressing endoderm (**J**), and alpha smooth muscle actin (αSMA)-positive mesodermal cells (**K**). Bar: 200 µm.

Cells isolated from a void urine sample of a pre-menopausal women (Supplemental Fig. S1) were reprogrammed using the integration-free epi5™ system. Several individual iPSC colonies were isolated, sub-cultured, expanded, and cryopreserved (Fig. 1A). The urine cell-derived iPSC line (uiPSC#1), hereinafter named U1, was further characterized and utilized in this study. U1 could be maintained undifferentiated for more than 30 passages while showing homogenous expression of the endogenous pluripotency markers OCT4, SOX2 and NANOG (Fig. 2A–G). We further assessed the expression of 31 pluripotency markers in U1 by RT-qPCR at passage 10 compared to the control female hESC line H9. The tested pluripotency markers are expressed in U1 at similar levels as compared to H9 in this qualitative comparison (Supplemental Fig. S2). Moreover, U1 reveals a normal female karyotype (Fig. 2H). Teratoma formation assay of U1 injected into immune-deficient mice shows trilineage differentiation capacity into ectodermal (TUBB3 positive), endodermal (HNF-3β positive), and mesodermal (αSMA positive) cells (Fig. 2I–K). This indicates successful reprogramming of urine-derived cells from female patient into iPSCs and their capacity for lineage-specific differentiation.

### Fibroblast differentiation from pluripotent stem cells

**Figure 3:**
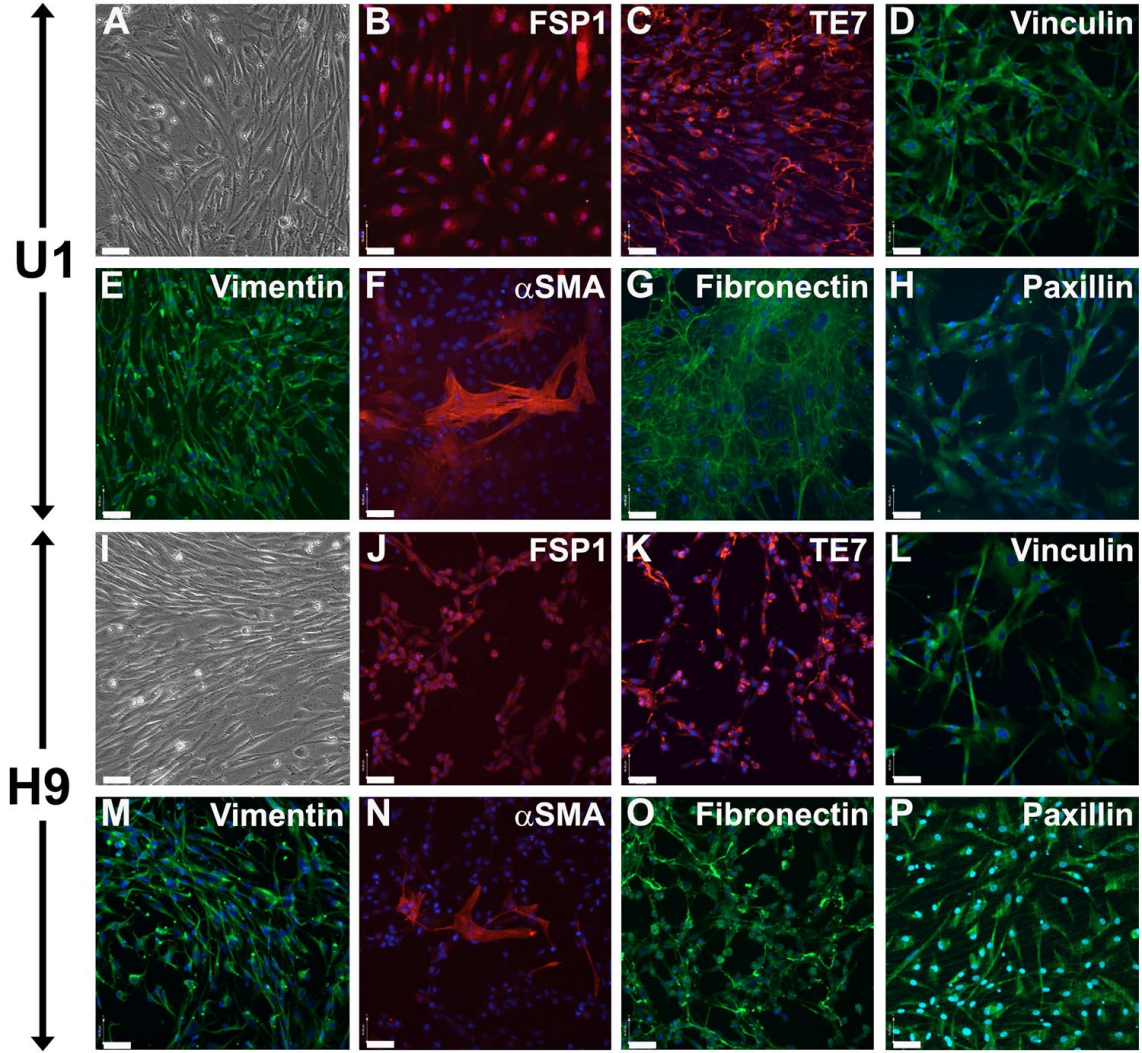
Characterization of U1 and H9-derived fibroblasts. After 5 passages in monolayer culture, de novo differentiated human fibroblasts from iPSC line U1 (**A**–**H**) and control hESC line H9 (**I**–**P**) were characterized by phase contrast (**A**, **I**) and immunofluorescence for anti-human fibroblast-specific antibody markers: fibroblast specific protein-1, FSP-1 (**B**, **J**); human thymic fibroblast marker, TE7 (**C**, **K**); vinculin (**D**, **L**); vimentin (**E**, **M**); alpha smooth muscle actin, αSMA (F, N); fibronectin (**G**, **O**); paxillin (**H**, **P**). Nuclear counterstain DAPI, bar: 40 µm.

De novo differentiation of fibroblasts from U1 and H9 was achieved via EB formation assay (Fig. 1B) as described before^20^. Single cell suspensions of undifferentiated U1 or H9 were aggregated to form uniform size EBs, then transferred to suspension culture in chemically defined E6 medium, supplemented with bFGF, where they grow is size and spontaneously differentiate. EBs were then enzymatically dissociated and seeded on collagen IV-coated culture dishes in fibroblast-promoting medium E6 supplemented with 10 ng/mL bFGF. Fibroblast enrichment and depletion of non-fibroblast cells occurred by repeated passaging. After five passages, U1 and H9 fibroblast (Fig. 3A, I) cells were characterized for the expression of fibroblast-specific markers and cryopreserved. Both, U1 and H9-derived fibroblasts show homogenous expression of fibroblast-specific protein-1 (FSP-1, Fig. 3B, J) and fibroblast-detecting antibody TE7 (Fig. 3C, K). Fibroblasts stain positive for intermediate filament vimentin (Fig. 3E, M), and individual cells exhibit spontaneous differentiation into αSMA-positive myofibroblasts (Fig. 3F, N). Cells show homogenous expression of extracellular marker fibronectin (Fig. 3G, O) and the focal adhesion molecules vinculin (Fig. 3D, L) and paxillin (Fig. 3H, P), which are involved in fibroblast migration.

To further assess purity of the de novo differentiated U1 fibroblasts, we immune-stained cultures with neuronal cell marker HNK-1, which is also expressed in subset of lymphocytes (human natural killer-1, CD57, Supplemental Fig. S3A). In the U1-fibroblast cultures, only individual cells (< 1%) stained positive for HNK-1. Moreover, they do not show presence of THY1(CD90)-positive mesenchymal stem cells (Supplemental Fig. S3B). Neuronal and muscle marker NCAM1(CD56) (Supplemental Fig. S3C), and smooth muscle myosin-heavy-chain (SM-MHC) expressing cells (Supplemental Fig. S3D) were absent from the U1 fibroblast cultures. Immunoblotting for HNK-1 and NCAM1 further proved absence of neuronal and muscle cells in U1-derived fibroblast cultures (Supplemental Fig. S3E). Both proteins were highly expressed in control tissue samples (brain, muscle), which confirms high purity of de novo differentiated U1-fibroblasts. We conclude that fibroblasts could be differentiated from iPSC lines in chemically defined culture conditions.

### Functional testing of fibroblasts

**Figure 4:**
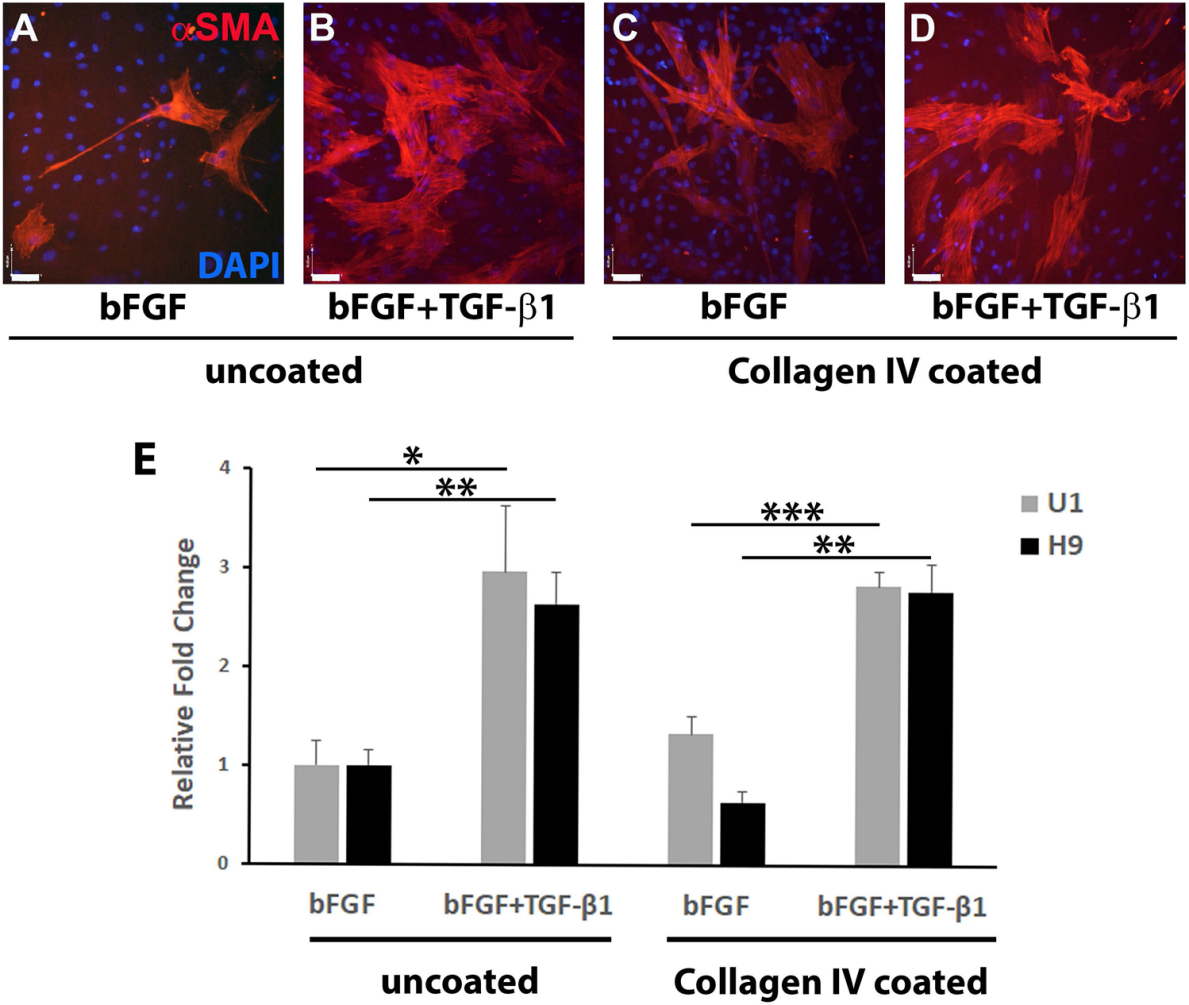
TGF-β1-induced myofibroblast differentiation of U1 and H9 fibroblasts. (**A**–**D**) Co-immunolabeling for αSMA and DAPI of fibroblasts cultured on: (**A**, **B**) uncoated, or (**C**, **D**) recombinant human collagen IV-coated plates; in (**A**, **C**) E6 culture medium with 10 ng/mL bFGF only; or (**B**, **D**) medium with 10 ng/mL bFGF + 2 ng/mL TGF-β1. Bar: 40 µm. (**E**) For each culture condition above (**A**–**D**), total numbers of DAPI-positive cells and αSMA-positive myofibroblasts, were counted in four visual fields. TGF-β1 treatment led to a significant increase of αSMA-positive myofibroblast differentiation as compared to untreated control cultures. In both, the U1 and H9 line, Collagen IV-coating did not have a significant impact on myofibroblast differentiation, independent of TGF-β1 supplementation. *p < 0.05, **p < 0.01, ***p < 0.001.

Fibroblasts can differentiate into myofibroblasts as part of developmental or wound-healing processes^21^, and TGF-β1 is a mediator causing resident tissue fibroblasts to differentiate into myofibroblasts as response to stressors^22^. Alpha smooth muscle action (αSMA, ACTA2) is a marker of mature myofibroblasts, which is expressed upon fibroblast activation and differentiation^21,22^. To assess the potential capacity of U1-fibroblasts to participate in would healing, we tested their responsiveness to TGF-β1 in vitro. U1- and H9-fibroblasts were cultured in E6 medium with bFGF on uncoated or collagen IV-coated dishes, and myofibroblast differentiation was induced by addition of 2 ng/mL TGF-β1. After 7 days of culture, the numbers of αSMA-positive myofibroblasts and the numbers of DAPI-stained nuclei were counted (Fig. 4A–D). Following TGF-β1 treatment, a significant 3.0 ± 0.7-fold increase in myofibroblast numbers was observed in U1-fibroblast cultures, and a 2.8 ± 0.2-fold increase was observed in H9-fibroblast cultures as compared to untreated cells. Collagen IV-coating had no effect on myofibroblast numbers in these cultures (Fig. 4E). This demonstrates the responsiveness of de novo differentiated fibroblasts to TGF-β1 and their capacity to differentiate into myofibroblasts upon stimulation.

### Myogenic differentiation of pluripotent stem cells and myocyte enrichment

**Figure 5:**
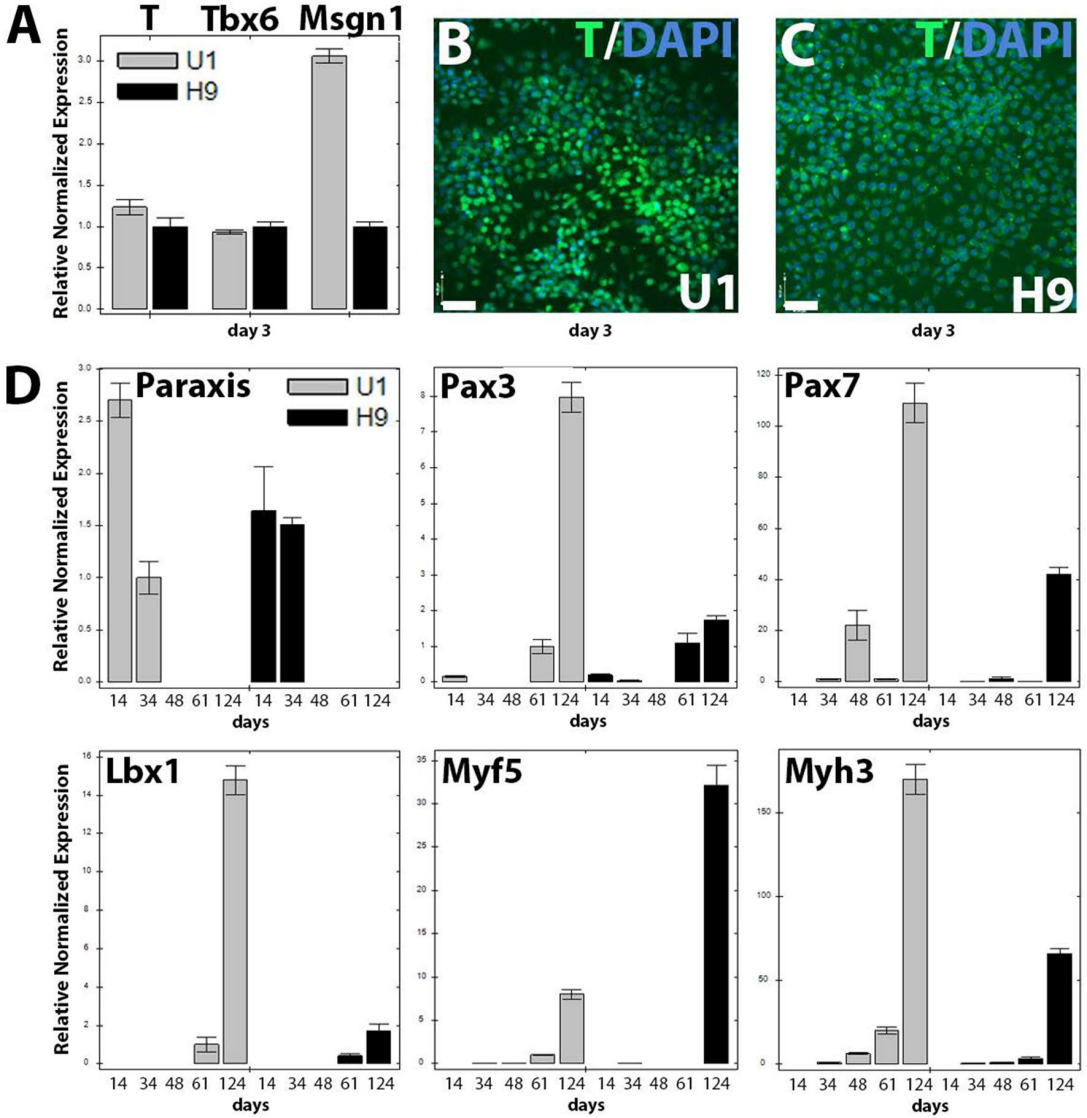
Mesodermal induction and myogenic differentiation of U1 and H9. (**A**) At day 3 of induction with CHIR99021, the early mesodermal transcription factors T (T-box T), Tbx6 (T-box 6), and Msgn1 (mesogenin 1) are detected by RT-qPCR. Immunolabeling for T antigen (nuclear counterstain with DAPI) reveals homogenous expression in (**B**) U1 and (**C**) H9 cultures at day 3. Bar: 80 µm. (**D**) RT-qPCR analysis of markers Paraxis, Pax3, Pax7, Lbx1, Myf5 and Myh3 reveals successful differentiation of U1 and H9 along the myogenic lineage into skeletal muscle progenitors and myocytes.

**Figure 6:**
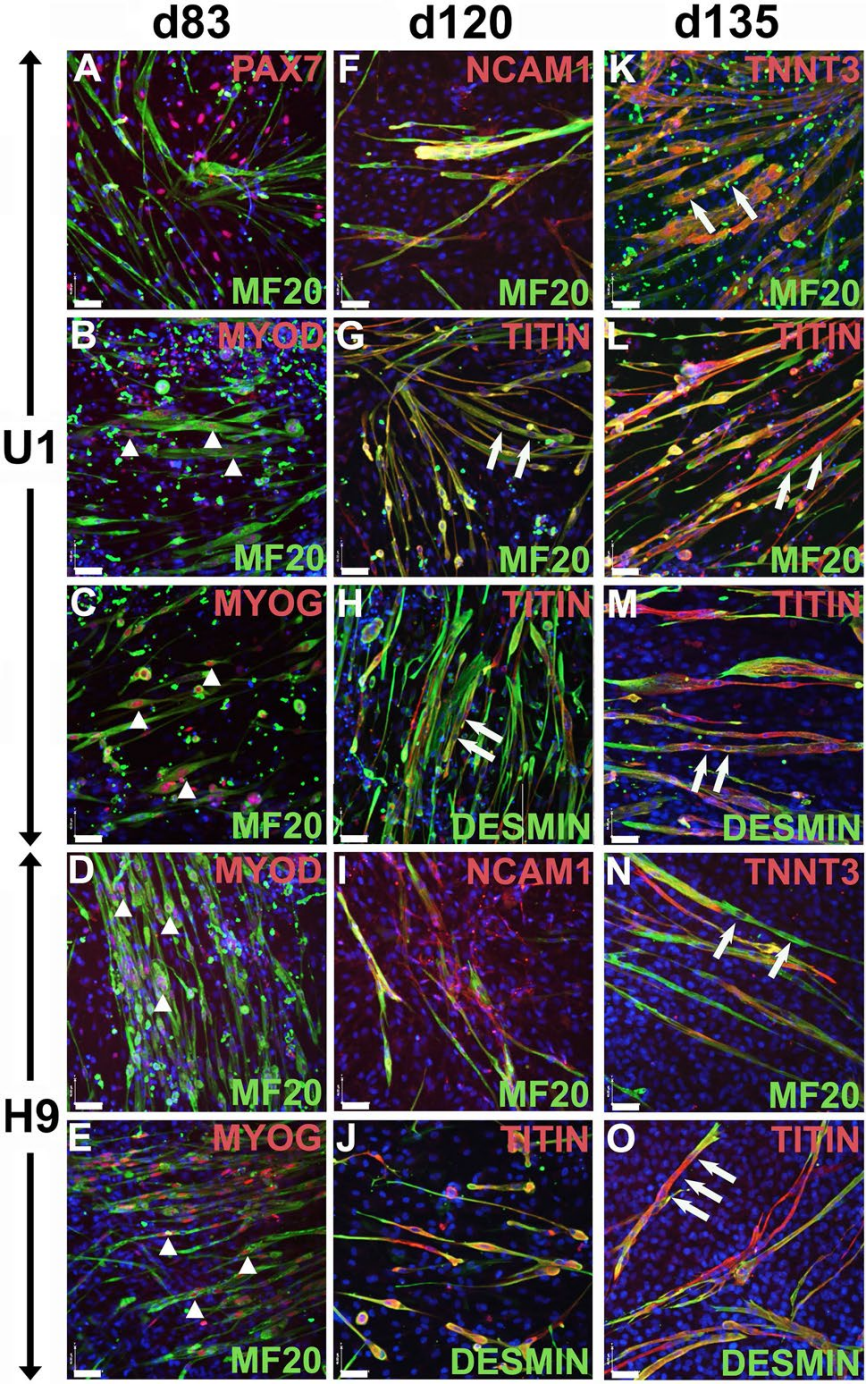
Characterization of U1 and H9 derived myocytes. Co-immunolabelling for myogenic lineage markers at days: d83 (**A**–**E**), d120 (**F**–**J**), and d135 (**K**–**O**) in differentiating cultures of U1 (**A**–**C**, **F**–**H**, **K**–**M**) and H9 (**D**, **E**, **I**, **J**, **N**, **O**). (**A**) At d83, U1 cultures show presence of cells expressing the myogenic progenitor marker PAX7 and cells expressing myocyte marker MF20. MF20 positive myocytes co-express MYOD (**B**, **D**, arrowheads) and MYOG (**C**, **E**, arrowheads). At d120, myocytes co-express MF20, NCAM1, TITIN or DESMIN (**F**–**J**). At d135, myocytes co-express MF20, TNNT3, TITIN or DESMIN (**K**–**O**). Multinucleated cells reveal myotube formation (**G**, **H**, **K**–**O**). Nuclear counterstain with DAPI, bar: 50 µm.

For de novo differentiation of skeletal muscle cells, we adapted three recently published protocols for mesodermal lineage differentiation of PSCs and their subsequent myogenic differentiation using serum-free culture conditions^23–25^. U1 and H9 were seeded in mTeSR1 medium in Geltrex-coated culture plates (Fig. 1C). The following day (day 0), mesodermal differentiation was induced by changing to E6 culture medium supplemented with 4 µg/mL of small molecule GSK3 inhibitor/WNT signaling inhibitor CHIR99021. Successful mesodermal lineage induction was confirmed by RT-qPCR at day 3, detecting the expression of transcription factor T (T-box T, brachyury), essential for mesoderm specification, and the early paraxial mesodermal transcription factors Tbx6 (T-box 6) and Msgn1 (mesogenin 1, Fig. 5A). Immunolabelling for T showed homogenous expression in U1 and H9 cultures at day 3 of the induction phase (Fig. 5B, C). From day 4 onwards cells were cultured in E6 + 10 µg/mL bFGF. bFGF is not essential for myogenic commitment but it stimulates proliferation and suppresses myogenic differentiation, allowing expansion of myogenic progenitors^23^. At day 40, cells were passaged and/or cryopreserved. Subsequent culture of cells on Geltrex in E6 medium (without bFGF) resulted in differentiation along the myogenic lineage into myocytes and myotubes. The process of myogenic differentiation up to day 124 was monitored by RT-qPCR analysis (Fig. 5D). The paraxial/somite marker Paraxis is detectable until day 34, followed by expression of myogenic precursor/satellite cell specific transcription factors Pax3, Pax7, Lbx1, myogenic regulation factor Myf5, and contractile muscle filament myosin heavy chain 3 (Myh3). This orchestrating cascade of overlapping skeletal muscle cell determining genes^26^ confirms successful skeletal muscle precursor differentiation from both U1 and H9 pluripotent stem cells. The increasing expression of the contractile muscle filament myosin heavy chain 3 (Myh3, Fig. 6D) reveals the presence of myocytes in the U1 and H9 cultures.

To further characterize the cells, and to evaluate the capacity of U1 and H9 to differentiate into skeletal muscle myocytes, we performed co-immunolabelling for myogenic proteins on day 83 and detected transcription factors PAX7, MYOD, or MYOG, as well as sarcomeric muscle filament MF20 (Fig. 6A–E). Transcription factor PAX7, which is required for specification of myogenic satellite cells, and muscle filament MF20 do not co-localize, indicating the presence of two myogenic progenitor cells as well as differentiated myocytes in U1 cultures (Fig. 6A). Accordingly, co-localization of MF20 with myoblast determining transcription factor MYOD (Fig. 6B, D), and MF20 co-localization with MYOG (myogenin, Fig. 6C, E) shows the presence of myoblasts and myocytes in the cultures. At day 120 (Fig. 6F–J) and day 135 (Fig. 6K–O) of myogenic differentiation, extensive formation of multi-nucleated myotubes (Fig. 6G, H, K–O, arrows) can be observed. Co-expression of MF20 with NCAM1 (Fig. 6F, I), TITIN (Fig. 6G, H, J, L, M, O), fast skeletal muscle troponin T3 (TNNT3, Fig. 6K, N), as well as co-expression of TITIN with DESMIN (Fig. 6H, J, M, O) indicates the presence of (elongating) skeletal muscle myocytes and myotubes in both, U1 and H9 cultures. This confirms the ability of the newly reprogrammed iPSC line to differentiate into skeletal muscle myocytes.

### Enrichment of skeletal muscle progenitors and myocytes

**Figure 7:**
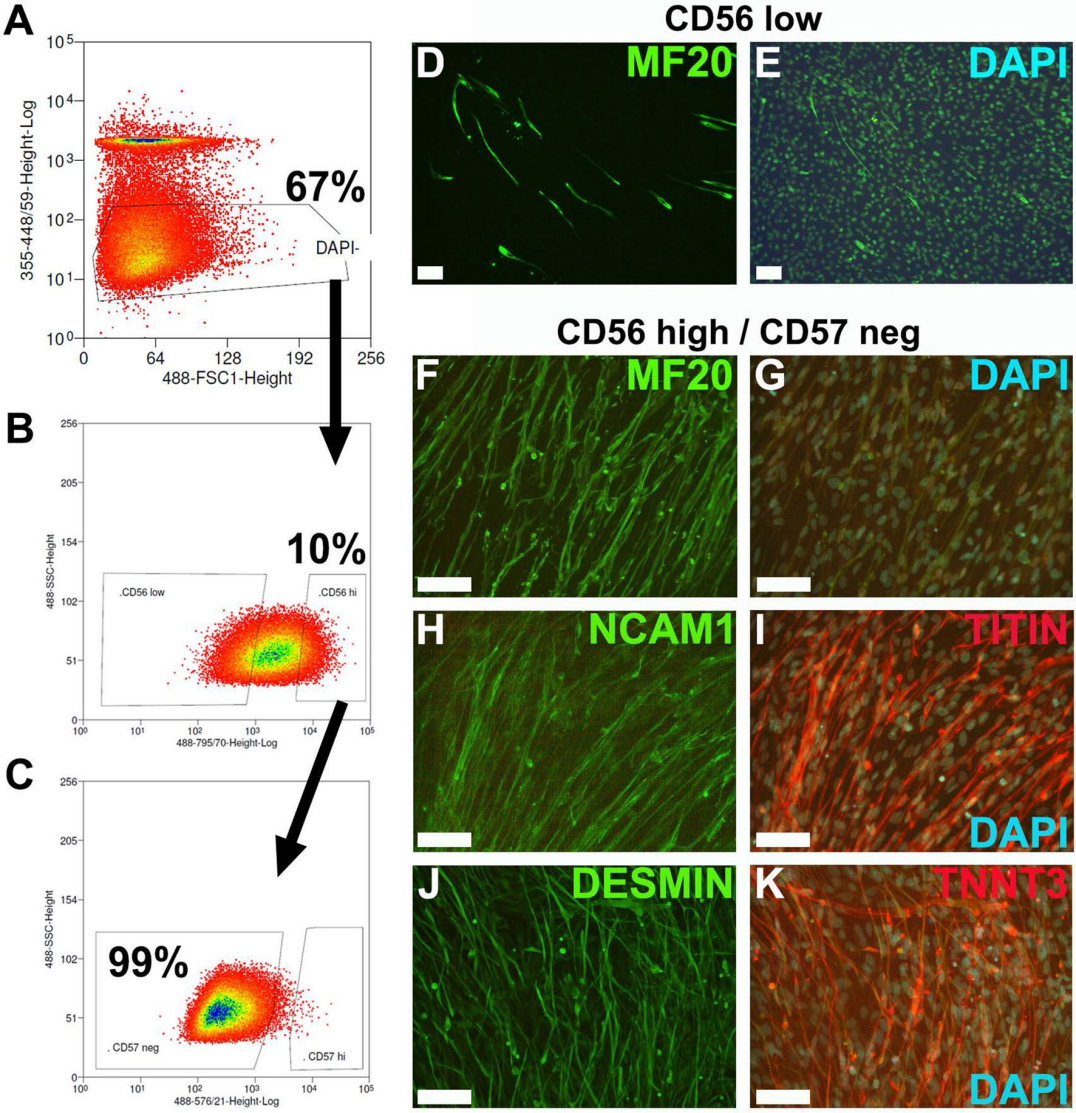
Enrichment of skeletal myocytes by fluorescence-activated cell sorting (FACS) using. Myogenic differentiation cultures of U1 (day 63) were dissociated and labelled with conjugated NCAM/CD56 and HNK-1/CD57 antibodies, and counterstained with DAPI. Gating Strategy: (**A**) DAPI-negative, intact cells were gated for CD56^high^ myocytes (**B**) and absence of CD57 expression (**C**). Sorted cells were cultured for 3 passages and labelled for markers. (**D**, **E**) Cultures of CD56^low^ cells show low numbers of myocytes, whereas cultures of CD56^high^/CD57^neg^ show myocyte enrichment as shown by expression of markers MF20 (**F**), NCAM1 (**H**), TITIN (**I**), DESMIN (**J**) and TNNT3 (**K**). DAPI (**E**, **G**, **I**, **K**) was used as counterstain. Bar: 100 µm.

Skeletal muscle progenitors and myocytes contribute around 20% of all cells in the differencing cultures. To enrich myoblast and myocytes, we performed FACS using the CD56/CD57 approach by Choi et al.^24^. Differentiating skeletal muscle cell cultures were enzymatically disaggregated after 50–60 days and labeled with conjugated CD56/NCAM1 and CD57/HNK-1 antibodies. The viable, DAPI negative cell population was sorted for cells with high expression of CD56 (Fig. 7A, B), which represented approximately 10% of total viable cells. Contaminations with neuronal cells were cleared by removal of CD57/HNK-1^pos^ cells from the CD56^high^ population (Fig. 7C). Sorted cell populations were cultured in E6 medium, expanded for 3 passages, cryopreserved, and tested for the expression of MF20. The CD56^low^ population (Fig. 7D, E) shows individual MF20 expressing cells, whereas the CD56^high^/CD57^neg^ population showed enrichment of MF20 expressing myocytes (Fig. 7F, G). Co-expression of NCAM1 and TITIN (Fig. 7H, I), as well as DESMIN and TNNT3 (Fig. 7J, K) further confirms enrichment of skeletal myocytes.

### Periurethral injection and tracing of fibroblast and myocytes in RNU female rats

**Figure 8:**
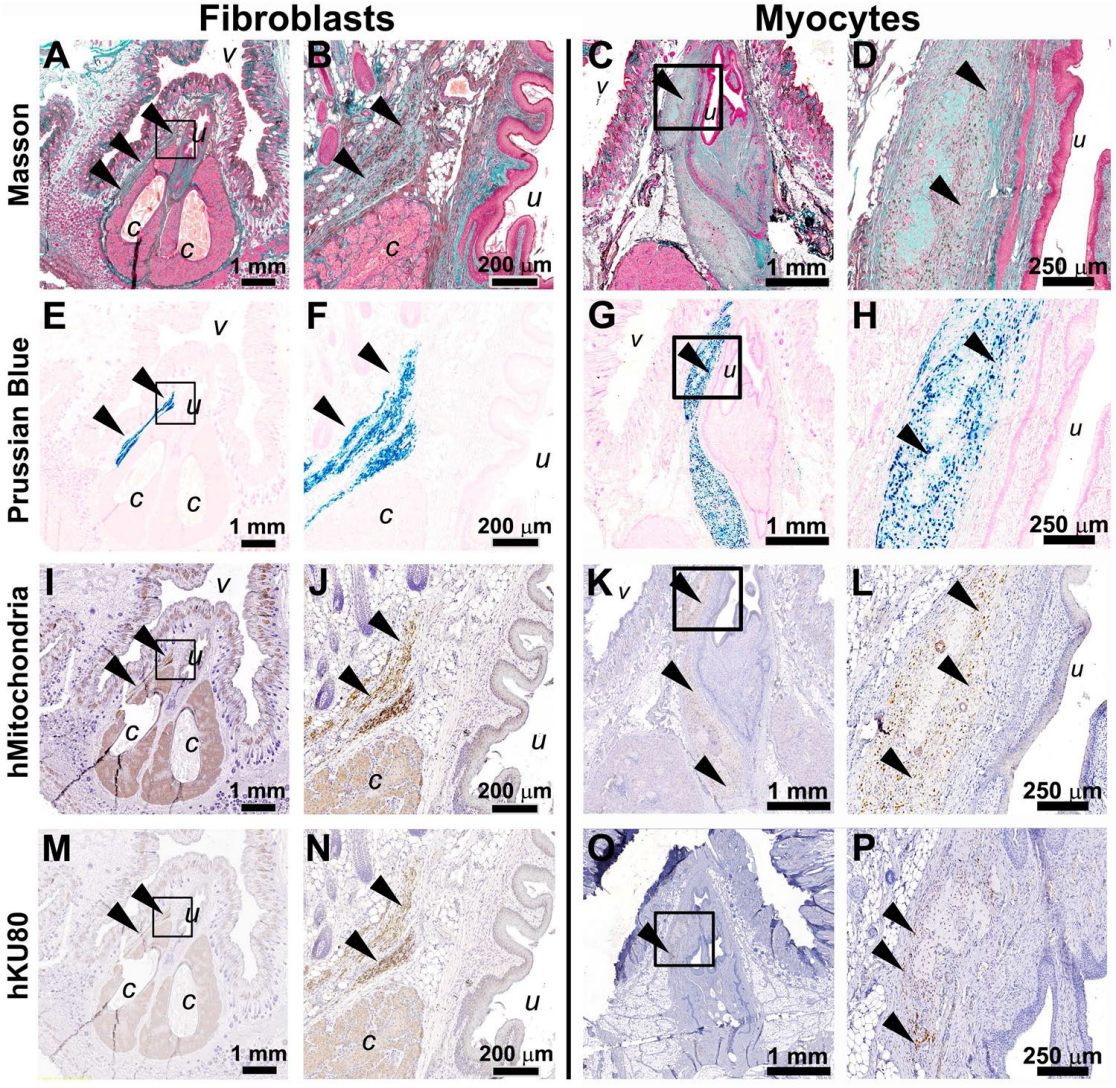
Histologic tracing of U1 fibroblasts and myocytes in the periurethral area of RNU rats. Histological images of the periurethral region of immune-compromised female nude rats injected with U1-derived fibroblasts (**A**, **B**, **E**, **F**, **I**, **J**, **M**, **N**) or U1-derived myocytes (**C**, **D**, **G**, **H**, **K**, **L**, **O**, **P**) at low and high magnification. (**A**–**D**) Masson’s trichrome histologic staining of the injection site (periurethral tissue area). (**E**–**H**) Prussian Blue staining (arrowheads) allows highly sensitive and specific detection of U1-fibroblasts (**E**, **F**) or myocytes (**G**, **H**) labelled with Molday ION Rhodamine B nanoparticles. (**I**–**L**) Immunohistochemistry staining with anti-human mitochondrial antigen antibodies, and (**M**–**P**) anti-human nuclear antigen KU80 antibodies confirm the presence of human fibroblasts or myocyte, respectively, within rat tissues (arrowheads indicate positive brown staining). *v*, vagina; *u*, urethra; *c*, clitoral gland.

We used immuno-deficient female RNU rats for engrafting of U1-fibroblasts and myocytes (Fig. 1D). For tracing of transplanted cells in rat tissue, they were labelled in vitro with the nanoparticle Molday ION Rhodamine B. Dissociated fibroblasts or myocytes were re-suspended in E6 medium: Matrigel mixture (50:50, 2*10^6^ cells in 100 µl) and injected into the peri-urethral area. Three weeks after injections, rats were euthanized, the urogenital tissues were dissected, cross-sectioned from the bladder neck to the urethral orifice, and Masson trichrome staining performed, demonstrating urethra, vagina, and clitoral glands with connective tissue collagen fibers (stained blue), epithelial keratins (dark red) and cellular cytoplasm (pink) (Fig. 8A–D). Prussian Blue staining was used to detect ferric iron in the Molday ION Rhodamine B tracer, incorporated into U1-fibroblasts or myocytes (Fig. 8E–H). This sensitive and specific labelling technique clearly shows presence of human cells transplanted into rat periurethral tissues and provides an easy-to-use tool for racing of transplanted cells. Furthermore, we used immunohistochemical staining with anti-human mitochondria specific antibody (Fig. 8I–L), and an anti-human nuclear protein KU80 antibody (Fig. 8M–P) labelling the same cell population as the Prussian Blue stain. Our results confirm persistence of labelled human cells in the periurethral region of female RNU rats and show: (i) de novo differentiated U1 fibroblasts and myocytes survive in the peri-urethral rat tissues for 3 weeks post-injection, and (ii) injected human cells can be tracked using Molday ION Rhodamine B ferric nanoparticles, and anti-human protein specific antibodies, (iii) iPSC-derived fibroblasts and myocytes provide a tool to test skeletal muscle and connective tissue regeneration in rat SUI models.

## Discussion

Pathophysiology of SUI is due to one of two problems; (i) urethral hypermobility and (ii) intrinsic sphincter deficiency^4^. In urethral hypermobility, the descent of the bladder neck and proximal urethra is caused by the weakness of the surrounding pelvic floor, which produces unequal pressure between bladder and proximal urethra, and thus, urine leakage occurs^5^. For correction of SUI, the midurethral support without tension was developed. Intrinsic sphincter deficiency occurs mainly from neuromuscular damage due to the loss of urethral tone that keeps the urethra lumen closed^4^. Sphincter weakness though is believed to be secondary to traumatic injury, neurogenic conditions and age-induced muscle degeneration^27^. Cell-based therapy holds great promise to restore or enhance the biological function of damaged urethral or pelvic floor tissue in mature women. With the development of iPSC technologies, it is now possible to generate many human cell types and consequently, the therapeutic potential of such technology for incontinence patients could be enormous. Regeneration of the sphincter complex using muscle-derived cells has been first demonstrated in animal models and displayed multiple qualities which provide advantage for treatment of sphincter deficiency^28^. In animal models of SUI, muscle-derived cells were incorporated into the damaged muscle and improved leak-point pressures on urodynamic characteristics^29^. Multiple clinical efforts were made to utilize human autologous myoblasts and fibroblasts for augmentation of urethral sphincter function^12^. Autologous cells derived from quadriceps femoris muscle biopsies of women with SUI and injected to repair urinary sphincter appeared safe^15,16^. In another study, minced autologous muscle tissue was used for intraurethral injection in women with uncomplicated and complicated SUI to repair urinary sphincter^17^. However, the process of cell isolation, expansion, harvesting, and engrafting is a complex, expensive and critical endeavor, dependent on the quality of culture cells at molecular level^30^.

The cellular component of the female urethra is composed of a combination of smooth and striated muscle cells^31^. Smooth muscle fibers are oriented both longitudinally and circularly, while the striated muscles that comprise the external urethral sphincter are in the middle portion of the urethra^31^. The extracellular matrix (ECM) component of urethral tissue is made from elastic and collagen fibers. The distinct muscular components of the urethra in combination with connective tissue provide a balance between extensibility and mechanical resistance of the sphincter to achieve its functioning for storage (continence). Studies have reported fragmented and disorganized elastic fibers and significant changes in collagen content in the periurethral tissues of women with SUI^32^, indicating an essential role of abnormal ECM homeostasis in the pathogenesis of female incontinence. Earlier work from Bertha Chen et al. tested a pure population of smooth muscle progenitor cells (pSMCs) derived from human PSCs of embryonic origin (H9) and from human iPSCs, which were transplanted into the urethra of the injury animal SUI model^7^. This group yielded histologic evidence of extracellular remodeling in the native rodent tissue and confirmed restoration of elastic fibers and muscle function after pSMC injections^7^. Moreover, their recent study suggests a potent paracrine effect of media conditioned by human PSC-derived pSMCs on improvement of function and histologic characteristics of the rat urethra and vagina^33,34^. These data suggest a novel therapeutic potential for PSC-based treatments for SUI and pelvic floor disorders where tissues are affected by loss of smooth muscle and ECM components collagen, or elastin.

Skeletal muscle component of the sphincter is external to the smooth fiber layers of the urethra in which fast striated fibers are prominent and could play a defining role in the mechanism of continence though phasic contractions^35^. To avoid the use of skeletal muscle tissue biopsies for obtaining autologous cells from SUI patients, we utilized an iPSC-based approach using cells from voided urine. We show here that live cells collected from voided urine can be propagated and used to derive iPSC with the episomal vector system, Epi5. Cell collection from voided urine is simple, painless, non-invasive and does not induce donor morbidity. Using this Epi5 system we have previously generated stable, transgene-free lines from human umbilical cord blood, MSCs, and fibroblasts but urine is the easiest to collect^19^. The advantage of the episomal vectors is that they do not integrate into the genome, therefore the risk of chromosome damage is limited; moreover, the nature of the episome is that it is eventually removed from all cells through dilution as it divides at a slower rate than cellular division leading to asymmetrical distribution in the cell population^36^. The iPSCs can easily be expanded and have the potential for direct differentiation into various cell types. For instance, episomal vectors are currently used in Japan to generate clinical grade iPSC for treatment of macular degeneration^37^.

Patient-specific iPSC from SUI patients can serve as a source for differentiation of autologous fibroblasts and muscle cells (both, smooth and striated muscle) for future treatment of the failing pelvic floor or urethral tissues. Our data show that iPSC-derived patient-specific skeletal myocytes, can potentially be used for the regeneration of the striated muscle tissues of the external sphincter. Several studies have shown that skeletal myoblasts can be differentiated from hiPSC and hESC^23,24,38^, which reveal normal electrophysiological properties^39^. These myoblasts can form striated contractile myofibers and myofibrils in vitro^24,39,40^ and also integrate into murine muscle myofibers in vivo^40^. This study demonstrates the potential value of the cell-based approach to regenerate striated muscle tissues in women with SUI to restore the function of the urethral sphincter^41^. Several rat models for SUI exist which allow testing of hPSC-derived human myocytes for integration into damaged urethral tissues, particularly into the striated muscle of the external sphincter^42^.

In addition, iPCS-derived fibroblasts could be used for autologous cell transplantation. Fibroblasts transplanted into the urethra can serve as a natural bulking agent, or become a part of the periurethral tissue, improving urethral function^43^. Fibroblasts are a dynamic cell type present in all tissues and organs, where they achieve selective differentiated states to mediate long-term tissue remodeling by regulating ECM production and acute wound healing^21^. Therefore, they are of high interest in the field of regenerative medicine and tissue regeneration. Fibroblasts can secrete growth factors to support tissue regeneration in coordination with inflammatory cells^44^. In response to tissue injury, mechanical stress, or cytokines, resting fibroblasts become activated and differentiate into migratory myofibroblasts. Migration triggered by injury exposes fibroblasts to new sources of signals that they are normally not subjected to, which could be conducive to fibroblast plasticity^44^. Once induced, myofibroblasts produce and secrete greater levels of ECM proteins, including multiple types of collagens, and express contractile protein, alpha-smooth muscle actin (αSMA), that underlie their ability to contract and close a wounded area^45^. In the present study we treated fibroblasts derived from iPSCs line U1, and from ESCs (H9 line) with TGF-β1, which caused the emergence of αSMA-positive myofibroblasts. The TGF-β family of growth factors is perhaps the most extensively studied mediator of fibroblast activation, of which TGF-β1 plays the greatest role^22^. Both, fibroblasts and myofibroblasts produce and maintain ECM of connective tissue layer supporting the anterior vaginal wall, and the periurethral tissue. Fibroblasts remodel their surrounding matrix by producing fibrillar proteins (collagen and elastin), and proteins responsible to biosynthesis and biodegradation of ECM. The balance in matrix production and catabolism influences the composition and mechanical properties of the connective tissue, maintains homeostasis of healthy tissue and provides a molecular basis for repair and regeneration of injured tissue^46^.

No animal model can fully simulate the multifactorial origin of SUI in humans; however, they are used for preclinical testing, improvement, and optimization of cell-based therapies for treatment of incontinence. In this study U1-fibroblasts were labeled with Molday ION Rhodamine B for in vivo tracking^47^ and injected into the periurethral area of female rats. We utilized immunodeficient Rowett Nude (RNU, NIH-Foxn1*rnu* athymic) rats to decrease adverse host immune reactions to injected human cells. Three weeks after injection, large numbers of engrafted human fibroblasts were detectable in the rat tissue, without signs of necrosis, immune-rejection or pathologic tissues invasion. We concluded that fibroblasts and skeletal muscle cells differentiated from patient-specific iPSC of SUI patients could be used for functional testing in rat models of SUI to evaluate their therapeutic potential in tissue remodeling. We showed that labelling fibroblasts and myocytes with Molday ION Rhodamine B is a very sensitive and specific method for cell tracing after periurethral injection.

We recognize limitations of this study as we generated iPSCs from voided urine samples of pre-menopausal women. The impact of gender and donor age on iPSC production and quality has not been extensively studied. Evidence suggests that cells from younger donors reprogram more efficiently compared to older donors but there is little data on quality differences of the final iPSC product^48^. Reduced efficiency of iPSCs production from old donors can be compensated by increased starting number of cells, however the question remains about the differentiation capacity of these cells. In the future we aim to generate urine-derived iPSCs from female patients with SUI regardless of their age/menopause status, which will allow us to compare their properties and therefore define the reprogramming efficiency.

In summary, we developed a workflow for the derivation of patient-specific iPSC that can be used to treat pelvic floor disfunctions. Our work demonstrates the feasibility of generating sufficient numbers of autologous fibroblasts and skeletal myocytes, from iPSC of female patients, avoiding invasive biopsies for cell isolations. Development of effective therapies to reduce SUI caused by intrinsic sphincter deficiency will have a dramatic impact in the future on the lives of the large proportion of mature women. We believe that this workflow can serve as a base for development of novel cell-based therapies for treatment of PFDs that overcome widely known problems associated with collection of autologous cells from patients.

## Supplementary Information

Supplementary Information 1.

Supplementary Information 2.

## Data Availability

All data generated or analyzed during this study are included in this published article and its supplementary information files.
