# Supplementary material for: Differentiation of patient-specific void urine-derived human induced pluripotent stem cells to fibroblasts and skeletal muscle myocytes
Source: Sci Rep. 2023 Mar 23;13:4746. doi: 10.1038/s41598-023-31780-9 (PMC10036466; doi:10.1038/s41598-023-31780-9)
Supplement: Supplementary file 1 — Supplementary Information 1. [file 41598_2023_31780_MOESM1_ESM.pdf]

Supplemental Table S1: Antibody list

**Primary antibodies**	**Company**	**Catalogue number**	**Host species**	**Clonality**	**Dilution**
OCT4	Thermo Fisher	PA5-27438	rabbit	polyclonal	1:100
SOX2	R&D Systems	MAB2018	mouse	IgG2a	1:100
NANOG	ReproCell	RCAB002P-F	rabbit	polyclonal	1:10
TUBB3	R&D Systems	MAB1195	mouse	IgG2a	1:100
HNF-3B	R&D Systems	AF2400	goat	polyclonal	1:100
αSMA	DAKO	M0851	mouse	IgG2a	1:100
FSP1	DSHB	CPTC-S100A4-3	mouse	IgG2c	1:50
TE7	Millipore	CBL271	mouse	IgG1	1:100
Vinculin	Sigma	V9131	mouse	IgG1	1:100
Vimentin	Santa Cruz	sc-7558	goat	polyclonal	1:50
Fibronectin	Santa Cruz	SC-8422	mouse	IgG1	1:150
Paxillin	BD Biosciences	612405	mouse	IgG1	1:100
T antigen (brachyury)	Santa Cruz	sc-17743	goat	polyclonal	1:100
PAX7	DSHB	PAX7-c	mouse	IgG1	1:200
MYOD	DAKO	M3512	mouse	IgG1	1:50
MYOG (myogenin)	Santa Cruz	sc-52903	mouse	IgG1	1:100
MF20	DSHB	AB2147781	mouse	IgG2b	1:200
NCAM1(CD56)	DAKO	M7304	mouse	IgG1	1:100
TITIN	DSHB	9D10-c	mouse	IgM	1:100
DESMIN	DAKO	M0760	mouse	IgG1	1:100
TNNT3	DSHB	JLT12-s	mouse	IgG1	1:100
hMitochondria	Millipore	MAB1273	mouse	IgG1	1:100
hKU80	Cell Signaling	2180	rabbit	polyclonal	1:100
HNK-1(CD57)	DSHB	1C10	mouse	IgG1	1:100
THY1(CD90)	Abcam	ab92574	rabbit	polyclonal	1:500
SM-MHC	Santa Cruz	sc-6956	mouse	IgG1	1:100
ERK2	Santa Cruz	sc-154	rabbit	polyclonal	1:1000
NCAM(CD56)-PE-Cy7	BD Biosciences	557747	mouse	IgG1	1:50
HNK-1(CD57)-PE	BioLegend	359612	mouse	IgM	1:50
**Secondary antibodies**					
Anti-Mouse IgG2a, Alexa Fluor 555	Thermo Fisher	A-21137	goat	polyclonal	1:400
Anti-Mouse IgG (H+L), Alexa Fluor 594	Thermo Fisher	A-11032	goat	polyclonal	1:400
Anti-Mouse IgG1, Alexa Fluor 568	Thermo Fisher	A-21124	goat	polyclonal	1:400
Anti-Mouse IgG1, Alexa Fluor 488	Thermo Fisher	A-21121	goat	polyclonal	1:400
Anti-Mouse IgG2b, Alexa Fluor 488	Thermo Fisher	A-21141	goat	polyclonal	1:400
Anti-Mouse IgM, Alexa Fluor 568	Thermo Fisher	A-21043	goat	polyclonal	1:400
Anti-Goat IgG (H+L), Alexa Fluor 488	Thermo Fisher	A-11055	donkey	polyclonal	1:400
Anti-Rabbit IgG (H+L), Alexa Fluor 488	Thermo Fisher	A-21206	donkey	polyclonal	1:400
Anti-Rabbit IgG (H+L) Alexa Fluor 568	Thermo Fisher	A-10042	donkey	polyclonal	1:400
**Western Blotting antibodies**					
Anti-Rabbit IgG horse radish peroxidase	GE Healthcare	NA934V	donkey	polyclonal	1:5000
Anti-Mouse IgG horse radish peroxidase	GE Healthcare	NA931V	sheep	polyclonal	1:5000
**Immunohistochemistry antibodies**					
Anti-Mouse IgG, biotinylated	DAKO	E0354	rabbit	polyclonal	1:300
Anti-Rabbit IgG, biotinylated	Vector Labs	BA-1000	goat	polyclonal	1:200
Anti-Goat IgG, biotinylated	DAKO	E0466	rabbit	polyclonal	1:300

Table S2: Primer assay information

**PrimePCR pluripotency markers**		**Myogenic differentiation**	
Target	Unique Assay ID	Primer name	Sequence
HPRT-1	qHsaCID0016375	T-FOR	GTACTCCCAATCCTATTCTGACAAC
B2M	qHsaCID0015347	T-REV	CATTCCAAGGCTGGACCAAT
GAPDH	qHsaCED0038674	Tbx6-FOR	GTGTCTTTCCATCGTGTCAAGC
TFRC	qHsaCID0022106	Tbx6-REV	TATGCGGGGTTGGTACTTGTG
TBP	qHsaCID0007122	Msgn1-FOR	AGTCAGGATGTCTGTCCAGC
ALPL	qHsaCID0010031	Msgn1-REV	AGGTAATTCCGGAGGGTGTG
CCNA2	qHsaCID0017452	Paraxis-FOR	AGGGCCACGGAGATGAGCCT
CDC42	qHsaCID0037936	Paraxis-REV	GGTCCCCCGGTCCCTACACA
CDK1	qHsaCID0036777	Pax3-FOR	AGCTCGGCGGTGTTTTTATCA
CHD1	qHsaCID0006750	PAX3-REV	CTGCACAGGATCTTGGAGACG
CHD7	qHsaCID0013442	Pax7-FOR	ACCCCTGCCTAACCACATC
CNOT3	qHsaCED0001495	Pax7-REV	GCGGCAAAGAATCTTGGAGAC
CRIPTO-1	qHsaCED0023686	Lbx1-FOR	CCACTTTCGCCGCTTCTTAGGG
KLF2	qHsaCED0005139	Lbx1-REV	ACGTTTAAGGGGCTGGAGGTC
DNMT3B	qHsaCED0042577	Myf5-FOR	AATTTGGGGACGAGTTTGTG
FGF2	qHsaCID0015510	Myf5-REV	CATGGTGGTGGACTTCCTCT
GDF3	qHsaCED0003639	Myh3-FOR	TTGATGCCAAGACGTATTGCT
KAT5	qHsaCID0020266	Myh3-REV	GGGGGTTCATGGCGTACAC
LIN28A	qHsaCID0022315	β-actin-FOR	GCATTGTTACAGGAAGTCCCTTG
NANOG	qHsaCED0023824	β-actin-FOR	CTATCACCTCCCCTGTGTGGA
PRDM14	qHsaCID0018592	HPRT-1-FOR	TGACACTGGCAAAACAATGCA
POU5F1 (OCT4)	qHsaCED0038334	HPRT-1-REV	GGTCCTTTTCACCAGCAAGCT
LEFTY2	qHsaCED0045158	SDHA-FOR	TGGGAACAAGAGGGCATCTG
SOX2	qHsaCED0036871	SDHA-REV	CCACCACTGCATCAAATTCATG
MYC	qHsaCID0012921	YWHAZ-FOR	ACTTTTGGTACATTGTGGCTTCAA
PAF1	qHsaCED0003366	YWHAZ-REV	CCGCCAGGACAAACCAGTAT
RIF1	qHsaCID0012192		
SALL4	qHsaCID0014611		
SMAD1	qHsaCED0037732		
SMAD2	qHsaCID0022031		
STAT3	qHsaCID0010912		
TERT	qHsaCID0009247		
THAP11	qHsaCED0019241		
TRIM28	qHsaCID0021255		
UTF1	qHsaCED0007920		
ZFP42	qHsaCID0013010		

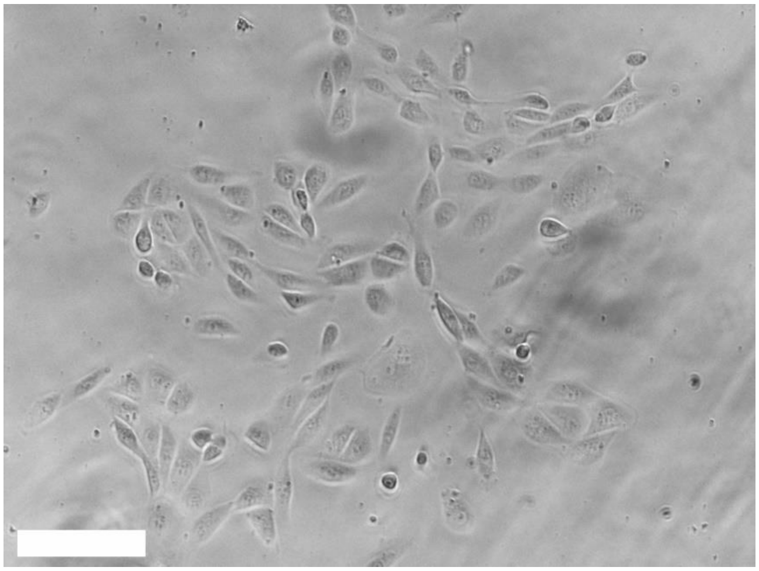

Figure S1. Cells collected from void-urine samples. Cells were collected by centrifugation from void-urine samples, plated into uncoated culture plates and cultured in Renal Epithelial Cell medium (ATCC, USA) without antibiotics. Phase-contrast image was taken at day 10 after initial seeding (P0). These cells were used for iPSC reprogramming and derivation of the U1 line. Bar: 100 µm.

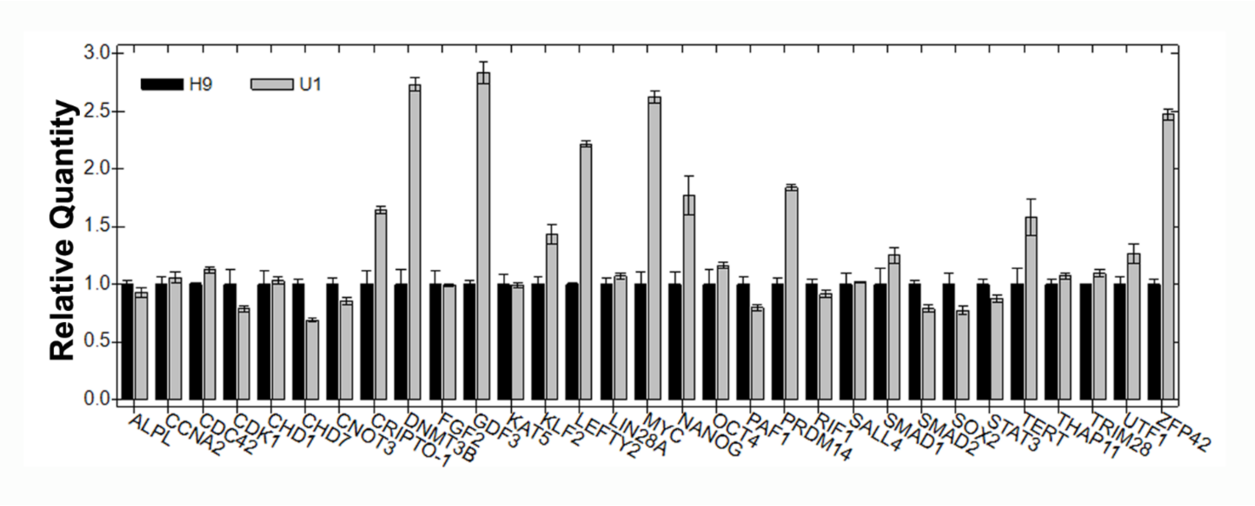

Figure S2. Real-time RT-qPCR analysis of U1 and H9. The expression of 37 pluripotency marker genes was tested at one parallel culture of hESC line H9 and iPSC line U1. Standard deviation shows variation of technical replicates. This qualitative analysis demonstrates that all tested pluripotency markers are expressed in the U1 line support immunofluorescence pictures for pluripotency markers in Fig. 1A-G. The hESC line H9 was used as a control to prove similar expression range. Differences in expression levels of individual markers may be passage or iPSC reprogramming related.

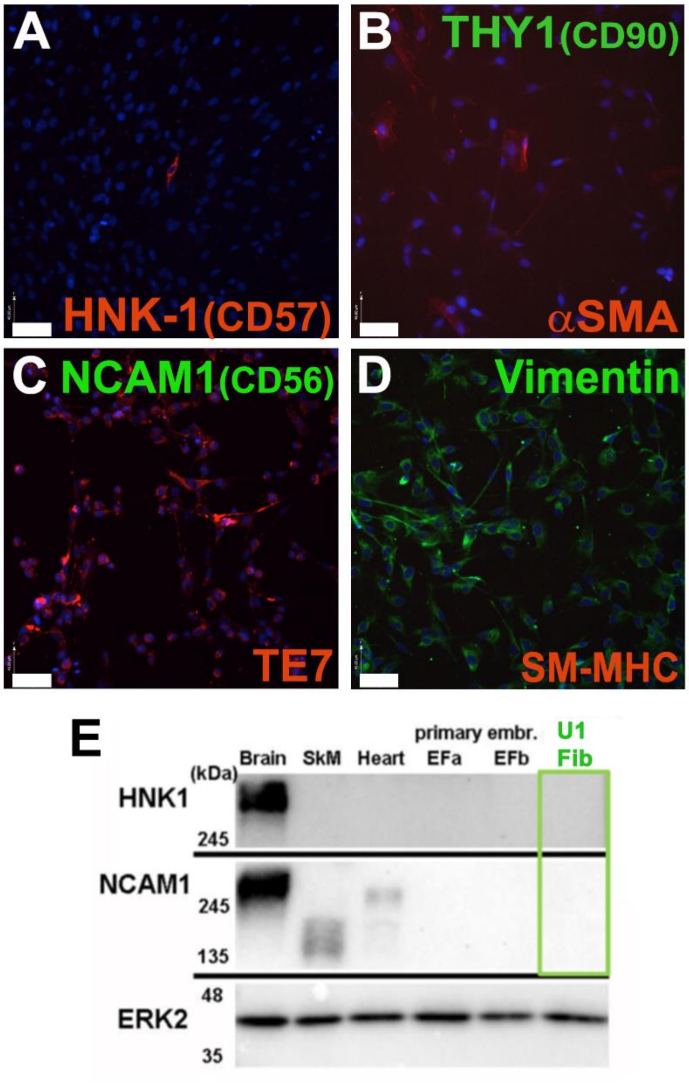

Figure S3. Purity analysis of *de novo* differentiated U1 fibroblasts. (A) Immunolabelling of U1 fibroblast shows only individual HNK-1(CD57)-positive neuroectodermal cells in the culture. (B) Co-immunolabeling shows absence of THY1(CD90)-expressing mesenchymal stem cells in fibroblast cultures, and presence of individual αSMA-positive myofibroblasts. (C) Co-immunolabeling shows absence of neuronal and striated muscle cell marker NCAM1(CD56), in TE7-expressing fibroblast cultures. (D) Cultures show uniform expression of intermediate filament vimentin but absence of smooth muscle myosin heavy chain (SM-MHC). Bar: 40 µm. (E)

Western Blot analysis confirming the absence of HNK1 and NCAM1 proteins, revealing high purity of the U1 derived fibroblasts cultures. 50 µg protein per sample were separated by electrophoresis using a 12% polyacrylamide gel and blotted onto a PVDF membrane (Bio-Rad). Membranes were incubated over night at 4°C with HNK-1, NCAM1 and ERK primary antibodies (Supplemental Table S1). After 3 washes, membranes were incubated with a horseradish peroxidase-conjugated secondary antibody, developed by Luminata ECL reagent (Millipore), and visualized on a Chemidoc instrument (Bio-Rad). U1 fibroblast cultures (green box) shows absence of HNK-1 and NCAM1 signals. ERK2 was used as loading control. Human fetal brain, skeletal muscle (SkM), and fetal heart lysates were used as positive controls. The human primary embryonic fibroblast lines EFa and EFb were used as negative controls.
