# Supplementary figures and images for: Differentiation of patient-specific void urine-derived human induced pluripotent stem cells to fibroblasts and skeletal muscle myocytes
Source: Sci Rep. 2023 Mar 23;13:4746. doi: 10.1038/s41598-023-31780-9 (PMC10036466; doi:10.1038/s41598-023-31780-9)

## Western-Blot Raw Data

NCAM1

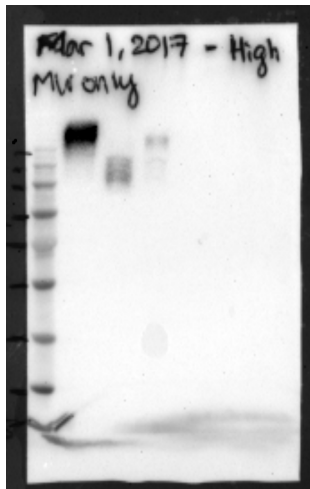

HNK1

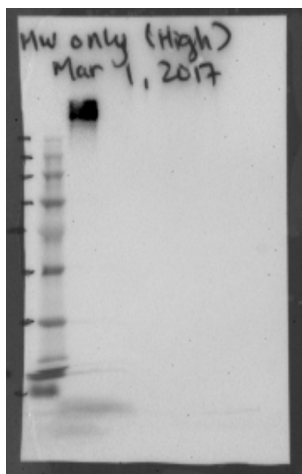

ERK2

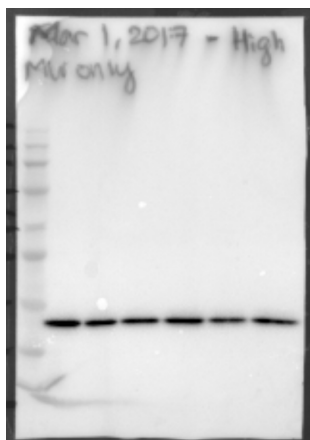

Supplement: Supplementary file 2 — Supplementary Information 2. [file 41598_2023_31780_MOESM2_ESM.pdf]
